# Assisted reproductive technique outcomes in patients with endometrioma undergoing sclerotherapy vs laparoscopic cystectomy: Prospective cross‐sectional study

**DOI:** 10.1002/rmb2.12386

**Published:** 2021-05-05

**Authors:** Saeed Alborzi, Elham Askary, Pegah Keramati, Shaghayegh Moradi Alamdarloo, Tahereh Poordast, Mohammad Ali Ashraf, Zahra Shomali, Behieh Namavar Jahromi, Ziba Zahiri Sorouri

**Affiliations:** ^1^ Department of Obstetrics and Gynecology Laparoscopy Research Center School of Medicine Shiraz University of Medical Sciences Shiraz Iran; ^2^ Department of Obstetrics and Gynecology Infertility Research Center School of Medicine Shiraz University of Medical Sciences Shiraz Iran; ^3^ Department of Obstetrics and Gynecology School of Medicine Shiraz University of Medical Sciences Shiraz Iran; ^4^ Department of Obstetrics and Gynecology Laparoscopy Research Center School of Medicine Guilan University of Medical Sciences Rasht Iran

**Keywords:** assisted reproductive technology, endometriosis, ethanol sclerotherapy, surgical management

## Abstract

**Purpose:**

The authors compared assisted reproductive technique (ART) outcomes and the recurrence rate of endometrioma in the infertile patients undergoing sclerotherapy vs laparoscopic ovarian cystectomy.

**Methods:**

In this prospective cross‐sectional study, a total of 101 infertile patients, with unilateral endometriomas, were divided into two groups. The first group (n = 57) underwent ART after 1 year of unsuccessful spontaneous pregnancy after laparoscopic ovarian cystectomy; the second group (n = 44) had ethanol sclerotherapy (EST) at the time of oocyte retrieval. The authors measured the number of oocytes, clinical pregnancy rate (CPR), live birth rate (LBR), complication, and recurrence of endometriomas as the primary and secondary outcomes.

**Results:**

The two groups had no significant differences in baseline characteristics and ovarian stimulation markers and also total number of oocytes. 42.1% and 34.1% of the patients (n = 24 and 15) had clinical pregnancy, and 38.6% and 29.5% (n = 22 and 13) had live birth following ART cycles in the surgery group and sclerotherapy group (*P* = .41, 0.34). The recurrence rates were 14.0% and 34.1% in the surgery and sclerotherapy groups (*P* = .017, *X* ^2^ = 5.67).

**Conclusions:**

Ethanol sclerotherapy can be a good alternative to surgery concerning the treatment of endometrioma; however, the recurrence of the disease in this group is significantly higher.

## Graphical abstract

Considering the almost equal number of pregnancy rate and live birth rate in surgery and ethanol sclerotherapy (EST) groups, it can be concluded that EST can be a good alternative to surgery concerning the treatment of endometrioma, particularly in the reproductive age women.

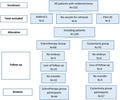

## 1 INTRODUCTION

Endometriosis is a prevalent chronic gynecologic condition associated with pelvic pain and infertility.^1^, ^2^, ^3^ Endometriomas diagnosed by ultrasound have been documented to have a persistent round shape and a thick wall cyst filled with low echogenic fluid.^4^, ^5^ Endometriosis was found in 25%‐40% of infertile women. Between 40% and 82.5% of the patients with endometriosis have ovarian endometrioma (OMA) concomitantly.^6^, ^7^, ^8^ According to the revised American Society for Reproductive Medicine (ASRM) classification, the presence of OMAs is more often associated with more advanced stages of the disease; its etiology and management, on the other hand, is still a matter of controvrsy.^9^, ^10^, ^11^, ^12^, ^13^, ^14^, ^15^

Despite its size, endometrioma can damage the ovaries by mechanical stretching. Its content leads to metaplasia and fibrosis and also reduces the cortical specific stromal cells as it contains inflammatory factors, proteolytic enzymes, and degrading agents.^16^, ^17^, ^18^ In addition, oxidative stress is much higher in the normal tissues surrounding endometrioma than it is in other benign ovarian cysts.^19^, ^20^, ^21^

Despite the high prevalence of endometriosis among infertile women, there have always been debates among gynecologists as to a treatment able to improve fertility, reduce pain, and prevent the recurrence of the disease. In other words, the most optimal treatment for OMA at reproductive age is yet to be known.^22^

Infertile women with endometriosis do not benefit from medical therapies. The main surgical procedures for treating endometriomas include laparoscopic cystectomy or fenestration and coagulation, ultrasound‐guided or laparoscopy‐guided aspiration, aspiration, and sclerotherapy.^23^ Laparoscopic ovarian cystectomy has been adopted as the method of choice because it is able to restore the normal anatomy, improve the pain, prevent recurrence, and increase the pregnancy rate, and it is also the only definitive approach to diagnosing endometriosis.^24^, ^25^ However, surgery is seemingly overestimated among endometriotic patients with infertility; this is while surgical operations would impact the ovarian function due to inadvertent removal or destruction of healthy ovarian tissues adjacent to the cyst wall; this in turn leads to the usage of higher gonadotropin doses to achieve follicular development in assisted reproductive technology (ART) cycles.^26^, ^27^

Imaging techniques are capable of detecting endometriosis lesions, which maintains the ovarian reserve until the end of reproductive ages for a complete surgery instead of multiple conservative surgeries; for OMA patients requiring ART, some authors have taken more conservative approaches such as aspiration and sclerotherapy, which could entail the disruption of the cyst epithelial lining with subsequent inflammation and fibrosis, eventually obliterating the cyst.^23^, ^28^, ^29^ They have also recommended sclerotherapy for recurrent endometriomas in patients that need ART or in symptomatic women who plan to conceive due to the chronic and persistent nature of the disease and the adverse effects of second cystectomy on ovarian reserve and fertility potential.^30^, ^31^

In the present study, we compared the ART outcomes in two groups of patients undergoing ethanol sclerotherapy (EST) at the time of ovum pickup prior to embryo transfer (ET) and those who could not conceive 1 year after laparoscopic ovarian cystectomy. Recurrence of endometriomas was also compared between the groups.

## 2 MATERIALS AND METHODS

### 2.1 Study population

This prospective cross‐sectional study, conducted from March 2013 to March 2020, included endometriosis patients referred to two private (Dena hospital) and university hospitals (Mother and child) affiliated to Shiraz University of Medical Sciences (SUMS). Our study was approved by SUMS Institutional Review Board under the code number: IR‐SUMS‐REC.1395.S100, and the ethical approval code was IR‐SUMS.MED‐REC.1395.03.

All patients were infertile and had 3‐6 cm, unilateral endometrioma, which were diagnosed by transvaginal ultrasound (TVUS). The patients were informed of the two methods prior to ET and that none had been proven to be superior yet. All participants gave their informed consent before participation. The exclusion criteria were two or more operations for endometriosis, low ovarian reserve, use of estrogen‐suppressing drugs such as oral contraceptive pills, danazol, or gonadotropin‐releasing hormone (GNRH) agonists 6 months prior to the study, >40 years of age, severe male factor infertility (OAT and teratozoospermia or sperm count < million/mL), the plan to use surrogate uterus, egg, or embryo donation, a cyst size of <3 cm or over than 6 cm, or bilateral OMA.

The patients were divided into two groups. The first group underwent ovarian cystectomy and removed all of deep infiltrative endometriosis (DIE) implants. In this group, ART was proposed if no spontaneous pregnancy occurred after 1 year. The second group comprised all patients who had endometriosis either without DIE or with small foci of DIE lesion. Regarding sclerotherapy, the second group directly went to the ART program while EST was performed at the time of ovum pickup. Fresh or freeze‐thawed embryo transfer (FET) was also carried out, and the patients were followed for any ultrasound sign of recurrence for up to 7 years. Maximum three cycles of ET were considered as the achievement of pregnancy.

### 2.2 Operation technique

Operative laparoscopies were conducted under general anesthesia by video control through a subumbilical incision (10 mm) and three or four lower abdominal incisions (5 mm). The utilized instruments were 5‐mm scissors and atraumatic graspers. Ovarian cystectomy was done after a sharp incision was made on the antimesenteric surface of the cyst, and traction and countertraction forces were applied using two atraumatic grasping forceps. The inner linings were sent for histologic examination. Hemostasis was achieved with Olympus bipolar device, and when necessary, irrigation was done with isotonic solution and suture to avoid the excessive use of bipolar energy. All adhesions were lysed and excised by sharp dissection to fully mobilize the ovaries. All areas of superficial active endometriosis, such as the other ovary or the pelvic peritoneum, were fulgurated. Endometriosis was classified according to the revised AFS classification.^9^ The patients' histologic reports were checked, and endometriomas were confirmed in all cases.

In the second group, at ovum pickup, vagina was prepared with povidone iodine solution under light general anesthesia in lithotomy position after the administration of 500 mg intravenous metronidazole and 1 m ceftazidime; afterward, it was washed thoroughly by saline solution to prevent the contamination of follicular fluid; oocytes were then retrieved by TVUS‐guided puncture needles. Next, endometrioma was drained and washed twice with normal saline 0.9%, once with ethanol 96% until the return fluid became clear, and one time at the end, where 80% of cyst volume ethanol was injected in the cyst and leave in. Aspirated chocolate material was sent for cytologic evaluation. After that, oral antibiotics were administered for 7 days. All operations were performed by the first author. Aspirated fluid and material of all patients revealed hemosiderin‐laiden macrophages, and they tested negative for abnormal neoplastic cells.

### 2.3 ART protocol

The antagonist protocol was selected for all the patients because the objective was to obtain the maximum number of ovum per cycle. A maximum dose of gonadotropin was also administered to patients with OMA to minimize the number of controlled ovarian hyperstimulation (COH) cycles.

Gonadotropins included recombinant follicle‐stimulating hormone (rFSH; follitropin alpha; Gonal‐f; Merck Serono) and human menopausal gonadotropin (HMG; Menogon; Ferring Pharmaceuticals), which were started on the second or third cycle days. After 5 or 6 days, TVUS was carried out to monitor the follicle response. Fixed dose of GnRH antagonist/day (Cetrotide, 0.25 mg; Merck Serpero) was injected to prevent the surge of premature luteinizing hormone (LH) until the trigger day and reduced the frequency of ultrasound examination sessions. Given the serum estradiol level and the risk of ovarian hyperstimulation syndrome (OHSS), the final oocyte maturation was triggered with human chorionic gonadotropin (10 000 IU of urinary HCG or 250 μg dose of rHCG; Ovitrelle, Merck Serono) or GnRH agonist (0.2 mg dose of triptorelin or diphereline; Decapeptyl; Ferring GmbH) once 2‐3 preovulatory follicles reached 17‐18 mm size. Ovum pickup (OPU) was done 36 hours later. For fresh ET, intramuscular (IM) progesterone injection (100 mg/day) was performed until the day of ET and then continued with cyclogest (400 mg, Actavis). Regarding frozen (FET) cycles, hormone replacement therapy (HRT) with estradiol valerate started from the second day of cycle until an appropriate endometrial thickness was reached (ET: 8‐9 mm); IM progesterone was then added and continued until 3‐5 days before ET (depending on the stage of embryo development); intramuscular progesterone was replaced by vaginal cyclogest pessary (400 mg, Actavis) throughout the luteal phase. Ultimately, to achieve pregnancy, a maximum of three ET cycles were performed during this study period.

### 2.4 Outcome measures

Assisted reproductive technique data were documented for each group, including the total dose and duration of gonadotropin therapy, serum estradiol level on the day of trigger, the quantity and quality of oocytes and embryos, the number of transferred embryos, and the clinical pregnancy rates (CPRs). Clinical pregnancy was considered as the detection of gestational sac within endometrial cavity by TVUS at 6 weeks of gestation. Throughout the follow‐up period, the two groups were compared regarding the recurrence of cyst as detected by TVUS.

Recurrence of the disease will be considered if we could detect a persistent round‐shaped, thick‐walled cyst, filled with low echogenic fluid, more than 2 cm in diameter in the previous affected ovary in ultrasound examination during the follow‐up period.^32^

Postoperation complications were recorded in the patient's chart.

### 2.5 Statistical analysis

Statistical analysis was performed using Fisher's exact test, chi‐square test, and paired sample t test. All the analyses were conducted by the statistical software package for Windows, version 16 (SPSS), and *P* values < .05 were considered as statistically significant.

## 3 RESULTS

**FIGURE 1:**
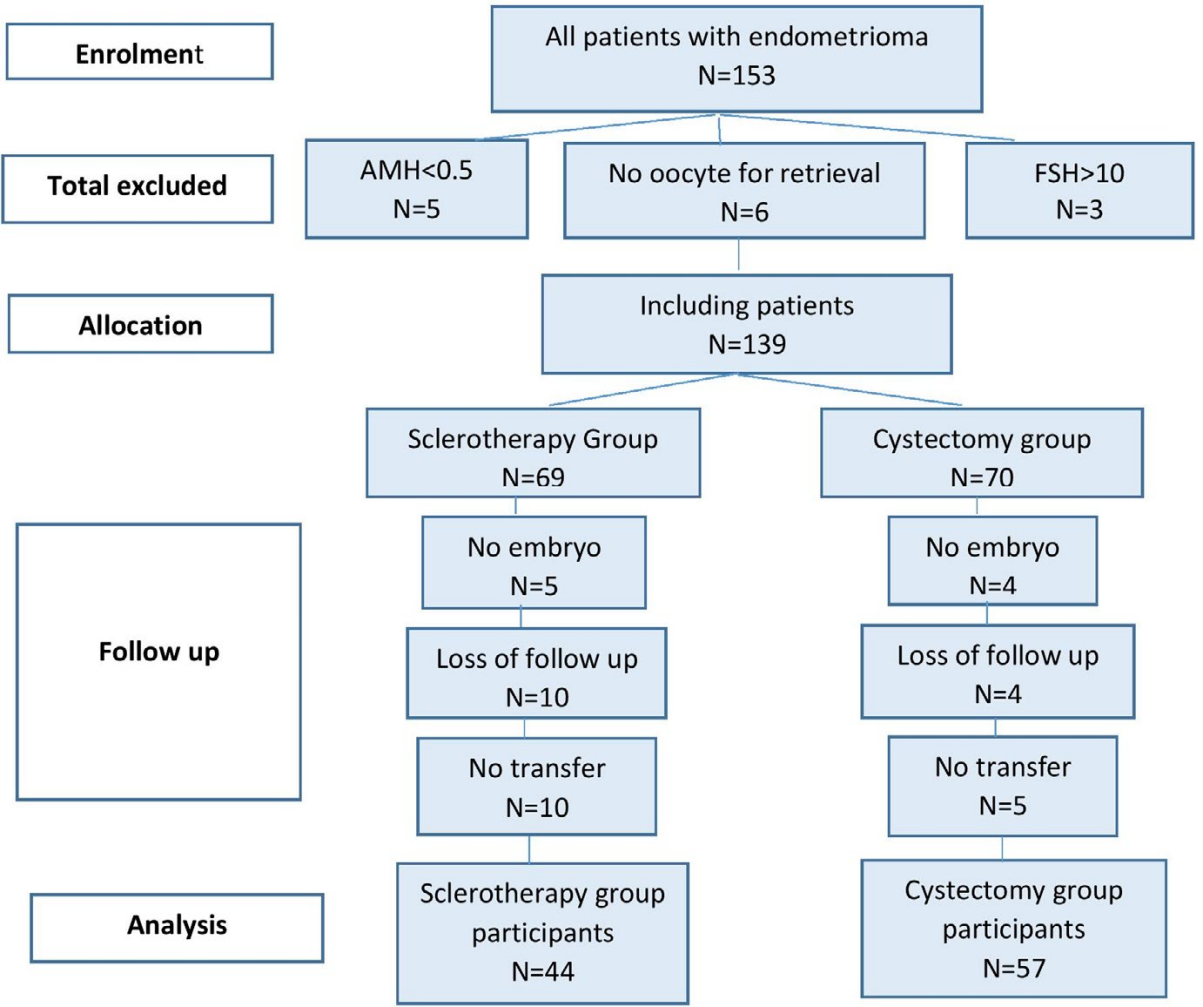
Illustrates study groups and numbers of patients per each group

Of 153 patients with endometriomas, eight patients had AMH < 0.5 or FSH > 10, and six had no oocyte for retrieval, hence excluded from the study. Five patients in the surgery group (group 1) and four in sclerotherapy group (group 2) had no embryos for transfer. Ten patients in group 1 and four in group 2 were lost to follow‐up; ten in the first group and five in the second group did not transfer the embryos over the study period. Finally, 57 patients (56.4%) in the surgery group and 44 patients (43.6%) in sclerotherapy group remained for analysis (Figure 1). Figure 1 illustrates the study groups and the number of patients per each group.

The baseline characteristics of both groups are shown in Table 1.

**TABLE 1:** Baseline Characteristics of the Patients

Variables (Mean ± SD)	Surgery group n = 57	Sclerotherapy group n = 44	*P*‐value
Age (y)	31.09 ± 3.95	30.8 ± 4.69	.74
BMI (kg/M^2^)	23.21 ± 4.4	23.77 ± 4.81	.54
Duration of Infertility (y)	2.73 ± 1.69	2.12 ± 1.41	.09
FSH (IU/mL)	7.58 ± 0.61	7.57 ± 0.64	.91
AMH (ng/mL)	2.71 ± 1.83	3.81 ± 3.91	.22
Size (CM)	4.52 ± 1.41	4.48 ± 1.51	.68

The two groups had no significant differences in baseline characteristics, which included age, BMI, duration of infertility, size of OMA, and baseline FSH and AMH levels.

Table 2 reports the ART outcome, where there was no significant difference between the groups.

**TABLE 2:** Comparing ART outcomes in both Groups

	Surgery group (n = 57)	Sclerotherapy group (n = 44)	*P*‐value
Total dose of gonadotropin (IU)	4375 ± 806.69	4544.12 ± 749.12	.35
Estradiol on the day of triggering (pg/mL)	1361.63 ± 355.03	1330 ± 577.71	.77
Duration of gonadotropin therapy	10 ± 1.39	10.08 ± 1.46	.78
Number of Oocytes < 3	17 (29.8)	6 (13.6)	.05
Number of total oocytes	6.11 ± 5.11	7.95 ± 5.18	.08
Number of MII oocytes	5.77 ± 4.03	6.66 ± 4.69	.31
Number of embryos	4.48 ± 3.1	5.18 ± 3.84	.32
Embryo A	3.53 ± 0.46	4.07 ± 0.72	.51
Embryo B	1 ± 0.25	1.28 ± 0.35	.51
Embryo C	0.1 ± 0.05	0.28 ± 0.84	.22
Fresh transferred patients	23 (54.8)	16 (45.7)	.43
Frozen‐thawed transferred patients	19 (45.2)	19 (54.3)	

The pregnancy outcome was also compared between the two groups (Table 3). We had 42.1% and 34.1% (n = 24 and 15) clinical pregnancy, and 38.6% and 29.5% (n = 22 and 13) live birth following ART cycles in the surgery group and sclerotherapy group, although the surgery group had higher CPR and live birth rate (LBR) compared to EST group but there is not statistically significant (*P* > .05).

**TABLE 3:** Comparison of pregnancy outcome in both group

	Surgery group (n = 57)	Sclerotherapy group (n = 44)	*P*‐value
Clinical pregnancy	24 (42.1)	15 (34.1)	.41
Birth rate	22 (38.6)	13 (29.5)	.34

During the follow‐up period, eight patients (14.0%) and 15 patients (34.1%) had OMA recurrence in the surgery and sclerotherapy groups, respectively, which shows a significant difference between the two groups (*P* = .017, *X* ^2^ = 5.67).

No case of tubo‐ovarian abscess (TOA) formation or malignancy was reported during the follow‐up.

Based on the results of multiple logistic regression analysis, no significant difference was found between the two groups in terms of pregnancy outcome after controlling for age, BMI, AMH, and number of embryos (*P* > .05).

The serum AMH level did not significantly change in sclerotherapy group during the follow‐up compared to the pre‐aspiration time.

## 4 DISCUSSION

In this study, surgery and EST procedures were compared in terms of ovarian reserve (AMH) and ART outcomes, recurrence rate, and complications during the seven‐year follow‐up in endometriotic infertile women. Due to the presence of OMA > 3 cm, all of our patients were categorized in stage III, IV ASRM.

Many studies have reported the negative effect of endometriosis and OMA on fertility.^26^, ^33^, ^34^, ^35^ In patients with bilateral OMA, women over 38 years, and patients with baseline AMH level < 2.5 ng/mL, ovarian cystectomy reduced follicular response in natural and clomiphene citrate‐stimulated cycles.^36^, ^37^ There is not strong evidence concerning routine endometriosis surgery before ART procedures, and only a small number of studies have reported that surgery is able to improve pregnancy outcomes following repeated IVF failure in endometriotic women.^38^, ^39^, ^40^ It is highly important to investigate other treatment methods of endometrioma such as EST owing to the reducing effects of surgery on ovarian reserve and the possibility of disease recurrence in reproductive ages, caused by the discontinuation in the medical suppression treatments.^15^, ^29^, ^30^

### 4.1 Ovarian reserve and ART outcomes

To the best of our knowledge, the present research is one of the very few prospective studies in this field with the longest follow‐up period (2‐7 years).

The most important aspect of this study was the similarity of baseline characteristics criteria between the two groups (Table 1). We found no significant difference regarding COH markers (total dose and duration of gonadotropins, peak level of E2, number of total and mature oocytes, number of embryos available for transferring, and quality of embryos) (Table 2) between two groups. Although the number of poor responder patients was higher in cystectomy group (n = 17, *P* value: .05), this group compared to EST group had a higher rate of CPR (42.1% and 34.1%) and LBR (38.6% and 29.5%); however, this difference was not statistically significant (*P* value: .41 and .34).

Similarly, Nickkho et al, in their systematic review, reported no difference between the surgery and control groups regarding CPR, number of oocytes, and gonadotropin dosage. They also reported the results of only four studies that compared the surgery and aspiration of OMAs without any difference in terms of CPR and LBR.^41^ Aflatoonian et al compared sclerotherapy and no treatment concerning the recurrence of OMAs after surgery. They found similar AFC, total oocytes, embryo, and CPR in both groups.^31^ In their systematic review and meta‐analysis, Cohen et al^42^ reported no difference in CPR between EST and cystectomy groups.

Contrary to our study, Lee et al compared three groups of endometrioma patients: surgical resection (n = 36), EST (n = 29), and no treatment group (n = 36); with consideration the low AFC count in the cystectomy group and the longer time duration between cystectomy and starting the ART methods compared to EST group from the beginning of the study (20.3 months vs 3.1 months, *P* < .001), response to COH was lower in the resection group than in two other groups; however, all three groups had similar clinical pregnancy (CPR), implantation, and miscarriage rates. By emphasizing this point that they did not mention, their patients underwent laparotomy or laparoscopy procedure for ovarian cystectomy.^43^

In their prospective study, Yazbeck et al made the same comparison between laparoscopic cystectomy and EST, but in patients with recurrence of OMAs. They reported higher ovarian response, oocyte pickup, and CPRs in EST group as it had higher AMH and lower FSH levels from the beginning of the research.^44^

Suganuma et al reported a reduced number of retrieved oocytes after the pretreatment of endometriomas with either laparoscopy/laparotomy resection or aspiration/ sclerotherapy vs no pretreatment before IVF; however, the fertilization rate was improved after cyst aspiration. They proposed that surgical pretreatment was not necessary for ovarian endometrial cyst before ART, but cyst aspiration might be beneficial after several failed attempts of IVF. They also concluded that the quality of oocytes did not change under the presence of OMAs.^45^

Hsieh et al reported an increase in AFC after EST, explaining that the decreased pressure on ovarian blood supply and the reduced mechanical pressure inside the ovary could improve the follicular growth after EST. By emphasizing this point that, they only concentrating on AFC and did not examine any other markers related to ovarian reserve before and after the procedure.^46^

We should mention here that the increased AFC after EST may result in better ultrasonic view contrary to the theory of increased ovarian function in the absence of OMA.^22^

We concluded that there was no difference between cystectomy and EST in terms of CPR, LBR, and other COH markers when both groups had similar baseline characteristics.

Therefore, in order to improve the fertility outcome, it seems that sclerotherapy with almost the same results as a surgery is a good choice to reduce pelvic inflammatory factors before ET in endometriosis patients. This method can also be used to avoid surgery, especially in cases of recurrent endometrioma during reproductive age.

### 4.2 Recurrence rate

The overall recurrence rate of endometrioma after EST ranged from 0% to 62.5%.^42^ The recurrence rate in EST group was significantly different when patients were followed for a longer period of time: 0%‐20% after 6 months of follow‐up to 11%‐28.6% after 16‐20 months.^31^, ^47^, ^48^, ^49^ In their recent meta‐analysis, Cohen et al^42^ reported that the risk of endometrioma recurrence in EST group was 3.47 times higher in the irrigation group compared with the retention group.

Recurrence rates for excision of endometrioma were reported to vary between 7.31% and 32% within 1‐6 years after excision.^15^, ^50^ Seo et al^51^ and Li et al^52^ reported that the recurrence rate of endometrioma after cystectomy was higher in patients with advanced endometriosis at the time of surgery and in younger patients; this rate varied from 22.5% in 30‐ to 39‐year‐old patients to 17.7% in 31‐ to 40‐year‐old patients at the time of operation. Based on the long follow‐up period of time of the patients in our study, the recurrence of endometrioma was much less than literate despite the advanced stage of disease in our patients.

In our study, the recurrence rate of OMAs after EST (34.1%) is similar to Agostini et al^49^ who reported 28.6% after 20 months of follow‐up; this rate is higher than most studies in the literature reporting 12% recurrence rate after ten‐month follow‐up,^44^ 14.9% after a 6‐month follow‐up [Noma and Yoshida, 2001^53^ in their retrospective study], and 11.1% after 16 months of follow‐up.^48^ This difference can be attributed to the longer follow‐up period in our study and the age of our study population (30‐31 Y/O).

In contrast to us, Garcia et al in their prospective cohort study reported that the recurrence of the endometrioma was higher in the surgical group (n = 14) than in the sclerotherapy group (n = 17); however, it was not significant. Here, it should be noted that in this study with a maximum follow‐up of 20 months, the number of patients with bilateral endometrioma in the surgical group was significantly higher than the sclerotherapy group from the beginning of the study (*P* = .004), which could explain the difference in recurrence rate between two groups. Also, the occurrence of pregnancy in the sclerotherapy group (three out of 17 cases) may justify less recurrence rate of disease in this group than in the surgical group.^54^

As was previously mentioned, sclerotherapy with 95% ethanol can be used as an appropriate alternative therapy to significantly reduce the pain in patients with recurrent endometriomas.^55^

### 4.3 Adverse outcomes

One of the advantages of cystectomy over sclerotherapy could be the former's more accurate diagnosis of ovarian lesions such as endometrioid cancer.^56^

However, we found no malignancy in all histologic samples obtained during cystectomy or sclerotherapy. Furthermore, the overall risk of malignancy arising from the endometriotic lesions was reported to be very low (0.8%‐0.9%), particularly in reproductive age with typical endometriotic features of cyst in ultrasonography.^57^, ^58^ Other risks associated with nonsurgical management could be as follows: rupture of the endometrioma and/or the development of a pelvic abscess, difficulties during oocyte retrieval, follicular fluid contamination with endometrioma content, progression of endometriosis, and post‐aspiration adhesion.^59^ However, based on Villettec et al^60^ tubo‐ovarian abscess following ART, procedure could be over reported. All said, TOA in endometriotic women (immediately or after ART) is best treated by appropriated intravenous antibiotics and surgery.^60^, ^61^, ^62^

None of the patients in our series developed serious complications.

### 4.4 Limitations

This study had a small number of patients in the sclerotherapy group, and there was no control group; these shortcomings give a call for future studies.

## 5 CONCLUSIONS

Considering the almost equal number of pregnancy rate and LBR in surgery and EST groups, it can be concluded that EST can be a good alternative to surgery concerning the treatment of endometrioma, particularly in the reproductive age women; however, the recurrence of the disease in this group is significantly higher. Therefore, performing surgery or sclerotherapy before IVF/ET should be individualized according to the patients' symptoms, signs, and desire for future fertility; in other words, repeated surgeries are to be avoided during the reproductive age with precise counseling regarding the currently available alternatives and accounting for the higher risk of recurrence in EST method.

## CONFLICT OF INTEREST

The authors declare that they have no conflict of interest.

## ETHICAL APPROVAL

Our study was approved by SUMS Institutional Review Board under the code number: IR‐SUMS‐REC.1395.S100, and the ethical approval code was IR‐SUMS.MED‐REC.1395.03.

## HUMAN RIGHTS STATEMENTS AND INFORMED CONSENT

All procedures followed were in accordance with the ethical standards of the responsible committee on human experimentation (institutional and national) and with the Helsinki Declaration of 1964 and its later amendments. Informed consent was obtained from all patients for being included in the study.
